# Sex differences in the neuronal transcriptome and synaptic mitochondrial function in the cerebral cortex of a multiple sclerosis model

**DOI:** 10.3389/fneur.2023.1268411

**Published:** 2023-11-02

**Authors:** Noriko Itoh, Yuichiro Itoh, Linsey Stiles, Rhonda Voskuhl

**Affiliations:** ^1^Department of Neurology, David Geffen School of Medicine, University of California, Los Angeles, Los Angeles, CA, United States; ^2^Department of Endocrinology, David Geffen School of Medicine, University of California, Los Angeles, Los Angeles, CA, United States; ^3^Department of Molecular and Medical Pharmacology, David Geffen School of Medicine, University of California, Los Angeles, Los Angeles, CA, United States

**Keywords:** multiple sclerosis, experimental autoimmune encephalomyelitis, sex differences, mitochondria, cerebral cortex, RNA sequencing, estrogen, sex chromosomes

## Abstract

**Introduction:**

Multiple sclerosis (MS) affects the cerebral cortex, inducing cortical atrophy and neuronal and synaptic pathology. Despite the fact that women are more susceptible to getting MS, men with MS have worse disability progression. Here, sex differences in neurodegenerative mechanisms are determined in the cerebral cortex using the MS model, chronic experimental autoimmune encephalomyelitis (EAE).

**Methods:**

Neurons from cerebral cortex tissues of chronic EAE, as well as age-matched healthy control, male and female mice underwent RNA sequencing and gene expression analyses using RiboTag technology. The morphology of mitochondria in neurons of cerebral cortex was assessed using Thy1-CFP-MitoS mice. Oxygen consumption rates were determined using mitochondrial respirometry assays from intact as well as permeabilized synaptosomes.

**Results:**

RNA sequencing of neurons in cerebral cortex during chronic EAE in C57BL/6 mice showed robust differential gene expression in male EAE compared to male healthy controls. In contrast, there were few differences in female EAE compared to female healthy controls. The most enriched differential gene expression pathways in male mice during EAE were mitochondrial dysfunction and oxidative phosphorylation. Mitochondrial morphology in neurons showed significant abnormalities in the cerebral cortex of EAE males, but not EAE females. Regarding function, synaptosomes isolated from cerebral cortex of male, but not female, EAE mice demonstrated significantly decreased oxygen consumption rates during respirometry assays.

**Discussion:**

Cortical neuronal transcriptomics, mitochondrial morphology, and functional respirometry assays in synaptosomes revealed worse neurodegeneration in male EAE mice. This is consistent with worse neurodegeneration in MS men and reveals a model and a target to develop treatments to prevent cortical neurodegeneration and mitigate disability progression in MS men.

## 1. Introduction

Multiple sclerosis (MS) is an autoimmune and neurodegenerative disease for which many disease-modifying treatments (DMTs) that target immune mechanisms exist. The discovery of neuroprotective treatments that target neurodegenerative mechanisms within the brain remains a goal. Genome-wide transcriptomics have shown differences in gene expression when comparing one brain region to another both when healthy and when affected by a disease, even within the same cell type, namely, neurons (1), microglia (2), astrocytes (3–5), and oligodendrocytes (6–8). Optimal therapeutic strategies aiming to reverse deleterious gene expression changes during disease may therefore need to be identified for distinct brain regions. One brain region of major interest in MS is the cerebral cortex. Cerebral cortex atrophy in MS correlates with worsening disability, as measured by the Expanded Disability Status Scale (EDSS) (9–11). Cortical pathology in MS includes neuronal damage and synaptic loss (12–17). A better understanding of neurodegeneration in the cerebral cortex at the molecular level is needed.

In addition to brain region-specific gene expression differences, when healthy and when affected by disease, there are also sex-specific differences. Sex differences can be mediated by biologic effects, environmental effects, or both. Observations that sex differences extend beyond humans, for example, to female and male mice in the same environment of a vivarium, reveal the importance of biologic effects. Biologic sex differences can be due to differences in sex hormones (estrogen vs. testosterone), sex chromosomes (XX vs. XY), or both (18, 19). Findings from the genotype-tissue expression (GTEx) project revealed the pervasiveness of sex differences in gene expression in humans. When 44 tissues were examined, 37% of all genes exhibited a sex bias in expression in at least one tissue (20). Whole-genome expression profiles showed distinct sex-biased regulatory networks that were tissue-specific (21), and sex differences in gene expression in the brain differed by the subregion (20, 22). These sex differences in gene expression in the human brain when healthy carry implications for sex differences in the brain when affected by a disease.

There are sex differences in MS (23). MS affects women more frequently than men (19, 24, 25). Despite the increased susceptibility of women, men with MS often demonstrate worse disability progression (26–29) and brain subregion atrophy (30–33). Thus, the effect of sex on inflammation vs. neurodegeneration appears to diverge. The female sex is more predisposed to develop autoimmune diseases, while the male sex appears to have a more neurodegenerative response to injury, reviewed in Voskuhl (34). Is there a model to disentangle these sex differences? Experimental autoimmune encephalomyelitis (EAE) is the most widely used MS model, and it was used to develop many of the current treatments for MS. Similar to MS, EAE is characterized by both inflammation and neurodegeneration. There are several EAE models, and the EAE model used depends on the MS question asked, as reviewed in Voskuhl (35). Relapsing remitting EAE in SJL mice shows an increase in susceptibility in female mice and is appropriate for the study of sex differences in immune responses in MS. EAE induced in SJL mice results in worse walking scores, spinal cord inflammation, and demyelination in female mice. In contrast, chronic EAE in C57BL/6 mice shows no sex difference in EAE walking scores or spinal cord pathology. Interestingly, the chronic EAE model exhibits brain atrophy as measured by *in vivo* magnetic resonance imaging (MRI) during later stages (35). This atrophy occurs in the cerebral cortex and cerebellum, and it is correlated with neuronal cell loss and a decrease in synapses (36–41). Cerebral cortex atrophy continues over time even after disability, as measured by standard EAE walking scores, has reached a plateau of severity. Such is also the case in MS patients whereby cerebral cortex atrophy by MRI continues even after disability, as measured by the EDSS, has reached a plateau of severity.

Here, we address whether there are sex differences in molecular mechanisms in the cerebral cortex during chronic EAE, focusing on transcriptomics of cortical neurons and mitochondrial respiration in synaptosomes.

## 2. Materials and methods

### 2.1. Ethics statement

All animal procedures were done in accordance with the guidelines of the National Institutes of Health and the Chancellor’s Animal Research Committee of the University of California, Los Angeles Office for the Protection of Research Subjects.

### 2.2. Animals and breeding strategy

All mice used in this study were from the C57BL/6 J background. Neuron Specific Enolase (NSE) Cre:RiboTag mice were generated by crossing the rNSEII-Cre line (42) with the RiboTag mice (43) (B6J.129(Cg)-Rpl22^tm1.1Psam^/SjJ, Jackson Lab, Bar Harbor, ME). In breeding of our mouse lines, Cre recombinase alleles were always inherited from female mice (mother) and not from male mice (father). Thy1-CFP-MitoS (44) mice generated by fusing the cyan fluorescent protein (CFP) construct with the mitochondria targeting sequence of human cytochrome C oxidase subunit 8 A (COX8A) under the regulatory elements derived from mouse Thymus cell antigen 1, theta (*Thy1*), were obtained from Jackson Lab (B6.Cg-Tg(Thy1-CFP/COX8A)S2Lich/J) for the visualization of neuron-specific mitochondria in C57BL/6 mice. The regulatory construct of *Thy1* gene defects in the sequence region was required for the expression in non-neuronal cells, making it specific to the neurons.

Genotypes were assessed by PCR using the following primer sequences (5′-3′): Cre, GCACTGATTTCGACCAGGTT and GCTAACCAGCGTTTTCGTTC; *LoxP-STOP-loxP-Rpl22-HA* (RiboTag), GGGAGGCTTGCTGGATATG and TTTCCAGACACAGGCTAAGTACAC; and Thy1-CFP-MitoS, AAG TTC ATC TGC ACC G and TCC TTG AAG AAG ATG GTG CG.

Animals were maintained under standard conditions in a 12-h dark/light cycle with free access to food and water *ad libitum*.

### 2.3. Active experimental autoimmune encephalomyelitis induction and clinical scoring

Mice were induced with active EAE as described previously for C57BL/6 mice (45). Briefly, mice aged 12–20 week were injected subcutaneously with myelin oligodendrocyte glycoprotein (MOG) amino acids 35–55 (200 μg/animal, Mimotopes, Richmond, VA) emulsified in Complete Freund’s Adjuvant, supplemented with *Mycobacterium tuberculosis* H37Ra (200 μg/animal, Becton, Dickinson and Company, Sparks, MD), over two sites drained by left inguinal and auxiliary lymph nodes in a volume of 0.1 mL/mouse. After 1 week, a booster immunization was applied subcutaneously over contralateral lymph nodes. Pertussis toxin (500 ng/mouse; List Biological Laboratories, Inc., Campbell, CA) was injected intraperitoneally on days 0 and 2 (46). The animals were monitored daily for EAE signs based on a standard EAE 0–5 scale scoring system: 0, healthy; 1, complete loss of tail tonicity; 2, loss of righting reflex; 3, partial paralysis; 4, complete paralysis of one or both hind limbs; and 5, premoribund state, as described (47).

### 2.4. Histological analysis

Standard histological analysis methods were applied as we described previously (42) with some modifications.

#### 2.4.1. Tissue preparation

Mice were deeply anesthetized in isoflurane and perfused transcardially with ice-cold 1x phosphate-buffered saline (PBS) for 5–7 min, followed by 4% paraformaldehyde for 5–7 min. Central nervous system (CNS) tissues were dissected and submerged in 4% paraformaldehyde overnight at 4°C, followed by 30% sucrose for >24 h. Brains were embedded in 7.5% gelatin/15% sucrose solution. The gelatin-embedded tissues were submerged in 4% paraformaldehyde overnight at 4°C, followed by 30% sucrose for >24 h, and stored at −80°C after flash frozen by dry ice. The 40-μm thick free-floating brain sagittal sections were prepared using a cryostat (Leica Biosystems, Nussloch, Germany) at −20°C. Tissues were collected serially and stored in 1xPBS with 0.1% sodium azide in 4°C.

#### 2.4.2. Immunohistochemistry

Prior to histological staining, 40-μm thick brain free-floating sections were thoroughly washed with 1xPBS to remove residual sodium azide. Tissues were incubated with 50% methanol/PBS (1:1) at RT, followed by 1xPBS washing. After heat antigen retrieval with heated 10 mM citric acid, 0.05% Tween 20, pH6.0, all tissue sections were permeabilized with 0.3% Triton-X in 1xPBS for 10 min on ice and blocked with 10% normal serum in 1x phosphate-buffered saline with Tween-20 (PBST) for 1 h. Tissues were then incubated with primary antibodies in 1xPBST-2% normal goat serum overnight at 4°C. The next day, tissues were washed and incubated with secondary antibodies in 1xPBST-2% normal serum for 2 h at RT. Sections were mounted on slides, allowed to dry, and cover slipped in fluoromount G (Southern Biotech, Birmingham, AL) for confocal microscopy. The following primary antibodies were used: rabbit anti-neuronal nuclear protein (NeuN; at 1:500 dilution, Cell Signaling Technology, Danvers, MA, cat#12943), rat anti-glial fibrillary acidic protein (GFAP; at 1:500, Thermo Fisher, Rockford, IL, cat# 13–0300), rabbit anti-ionized calcium-binding adapter molecule 1 (IBA1; at 1:1000, Wako Chemicals United States Inc., Richmond, VA, cat# 019–19,741), rabbit anti-glutathione S-transferase pi (GST-pi; at 1:1000, Enzo Life Sciences, Farmingdale, NY, cat#ADI-MSA-102,), mouse anti-parvalbumin (PV; at 1:1000, Swant, Burgdorf, Switzerland, cat# PV235), mouse anti-hemagglutinin (HA; at 1:500, BioLegend, San Diego, CA, cat#901522), and rabbit anti-HA (at 1:1000, Thermo Fisher Scientific, Rockford, IL, cat#MA5-27915). The following secondary antibodies were used at 1:500 dilution for staining the tissues: goat anti-rabbit Alexa Fluor Plus 647 (Thermo Fisher Scientific, Rockford, IL, cat#A32733), goat anti-rat Cy5 (Jackson Immunoresearch, West Grove, PA, cat# 112–175-167), goat anti-mouse- Cy5 (Jackson Immunoresearch, West Grove, PA, cat# 115–175-166), goat anti-rabbit TRITC (Jackson Immunoresearch, West Grove, PA, cat# 111–025-144), and goat anti-mouse- Cy3 (Jackson Immunoresearch, West Grove, PA, cat#115–545-166).

#### 2.4.3. Confocal microscopy and image analysis

Stained sections were examined and imaged using Olympus BX61 DSU fluorescence microscope with a Hamamatsu ORCA-FLASH4.0LT+ SCMOS CAMERA. NeuN and Thy1-MitoCFP images were captured at 243.75 × 243.75 um^2^ with 0.2 μm thickness of 20 stacks. Three images at the cortical region (bregma −3 to 0 mm/ lateral 0 to 1.2 mm/ Layer II-V) have been taken from one animal. All images were taken and processed using the integrated software program Olympus cellSens software version 4. ImageJ (NIH) was used to perform integration and analysis of images.

### 2.5. RNA co-immunoprecipitation, sequencing, and analysis

Mice were exposed to a lethal dose of isoflurane and transcardially perfused with ice-cold 1x PBS for 3–5 min, followed by ice-cold 1% paraformaldehyde-1x PBS for 4–5 min. Cortex were collected and snap-frozen in liquid nitrogen. Tissues were stored at −80°C. Frozen tissues were homogenized on ice using a dounce homogenizer with freshly made homogenization buffer containing: 50 mM Tris–HCl (Invitrogen, Carlsbad, CA) pH 7.5, 100 mM KCl (Thermo Fisher Scientific, Rockford, IL), 12 mM MgCl_2_ (Thermo Fisher Scientific, Rockford, IL), 1% Nonidet P-40 (Roche, Mannheim, Germany), 1 mM Dithiothreitol (DTT; Sigma-Aldrich, St. Louis, MO), 1× Proteinase Inhibitors Cocktail (Sigma-Aldrich, St. Louis, MO), 200 units/mL RNAsin (Promega, Madison, WI), 100ug/ml cycloheximide (Sigma-Aldrich, St. Louis, MO), and 1 mg/mL heparin (Sigma-Aldrich, St. Louis, MO). The homogenates were then centrifuged at 20,000× g, 4°C for 15 min. The supernatant was collected and incubated with pre-washed anti-HA conjugated magnetic beads (Pierce, Rockford, IL) overnight on a rotating wheel at 4°C. After removal of the supernatant, the magnetic beads were washed three times with high salt buffer containing 50 mM Tris (pH 7.5), 300 mM KCl, 12 mM MgCl_2_, 1% Nonidet P-40, 1 mM DTT, and 100 μg/mL cycloheximide. Then, 25 μL of proteinase K (4 mg/mL; Zymo Research, Irvine, CA) was added to the samples and incubated in a 55°C water bath for 30 min. After incubation, 300 μL tri-reagent was added, and the Direct-zol^™^ RNA MiniPrep Plus kit (Zymo Research, Irvine, CA) was used for RNA isolation according to manufacturer’s instructions. RNA quantity and quality were measured using the NanoDrop 2000 spectrophotometer (Thermo Fisher Scientific, Rockford, IL) and Agilent High Sensitivity RNA ScreenTape System (Agilent, Santa Clara, CA).

The RNA sequencing library was made using the KAPA Stranded RNA-Seq Kit (Kapa Biosystems, Wilmington, MA) which consists of mRNA enrichment, cDNA generation, end repair, A-tailing, adaptor ligation, strand selection, and PCR amplification. Barcoded adaptors were used for multiplexing samples in one lane. Sequencing was performed on Illumina HiSeq3000 for a paired-end 2x50 run. Data quality check was done on Illumina SAV. De-multiplexing was performed with the Illumina Bcl2fastq2 v 2.17 program. These procedures were performed at the UCLA Technology Center for Genomics and Bioinformatics core facility.

Sequencing analyses and production of figures were performed in R (R Core team, 2023).^1^ Qualities of raw sequence data were examined using FastQC,^2^ and Trimmomatic (48) was used for cleaning. The R package “QuasR” (49) was used for the read alignment to the mouse genome (mm10) followed by counting at the gene level. The genes with count numbers less than 1 in more than half of the samples were filtered out. Differentially expressed genes between male control and male EAE, as well as between female control and female EAE mice, were identified with R package “edgeR” (50). A false discovery rate of 0.1 was used as the threshold of differentially expressed genes. Canonical pathway enrichment analysis was performed for differentially expressed genes using ingenuity pathway analysis (QIAGEN, Redwood City).^3^ GO (gene ontology) enrichment analysis was performed using the package “clusterProfiler” in R (51).

### 2.6. Mitochondrial morphology analysis

Quantitative analysis of mitochondrial morphology was conducted using the Mitochondria analyzer plugin for ImageJ (52, 53).^4^ The plugin’s thresholding settings were adjusted as follows: subtract background at 1.25, sigma filter plus at 2, enhance local contrast at 1.4, and block size at 1.05 with a C-value of 11. Subsequently, a comparison was made between the number of mitochondria per volume and the average volume and diameter of mitochondria in both the control and EAE groups.

### 2.7. Isolation of cortical synaptosomes

Mice were deeply anesthetized with isoflurane and perfused transcardially with ice-cold 1× PBS for 1–2 min. Cerebral cortices were dissected out and subjected to a synaptosome isolation method as described by Dunkley et al. (54) and Flynn et al. (55) with slight modifications. Briefly, the cortex was rapidly isolated and rinsed with ice-cold sucrose/EDTA buffer (320 mM sucrose, 1 mM EDTA, and 5 mM HEPES, pH 7.4). It was then transferred to a pre-chilled Dounce glass homogenizer containing 1 mL of homogenizing buffer (320 mM sucrose, 1 mM EDTA, 5 mM HEPES, and 0.25 mM DTT, pH 7.4) and gently homogenized by 10 strokes with pestle A first, followed by 10 strokes with pestle B after the addition of an additional 1 mL of homogenizing buffer. The homogenate was then centrifuged at 1,200 g for 10 min at 4°C. The supernatant was carefully layered on top of a discontinuous Percoll gradient (3, 10, 15, and 23% Percoll in homogenizing buffer) in a 10 mL centrifuge tube and centrifuged at 31,000 g for 5 min at 4°C using a JA-25.50 fixed-angle rotor in a Beckman Avanti J-25 high-speed centrifuge. Synaptosomes were isolated and recovered from the band between the 10 and 15% and the 15 and 23% Percoll layers (Supplementary Figure 1A). The isolated synaptosomes were washed with ice-cold sucrose/EDTA buffer and then diluted into an ionic medium (20 mM HEPES, 10 mM D-Glucose, 1.2 mM Na_2_HPO_4_, 1 mM MgCl_2_, 5 mM NaHCO_3_, 5 mM KCl, and 140 mM NaCl, pH 7.4) for the measurement of respiration. The final synaptosome pellet was then resuspended in an ionic medium, and the protein concentration was measured by the BCA Protein assay (Cat# A53226, Thermo Fisher Scientific, Rockford, IL).

### 2.8. Seahorse respirometry assay

The respirometry assay was performed in the Mitochondrial and Metabolism Core at UCLA. For monitoring respiration, freshly isolated synaptosomal protein in the ionic medium was aliquoted into a 96-well microplate (Seahorse Bioscience, North Billerica, MA) coated with a 1:15,000 diluted polyethyleneimine (PEI). For intact respirometry, 8 μg per well of synaptosomal protein was loaded; for permeabilized synaptosomes, 6 μg per well was loaded for complex I substrates (pyruvate and malate) and 4 μg per well for complex II substrate (succinate with rotenone). The plate was centrifuged at 3,400 g for 20 min at 4°C. Then, the medium was replaced with 175 μL Seahorse incubation Medium [3.5 mM KCl, 120 mM NaCl, 1.3 mM CaCl_2_, 0.4 mM KH_2_PO_4_, 1.2 mM Na_2_SO_4_, 10 mM Glucose, 2 mM Pyruvate, 2 mM MgSO_4_, 5 mM HEPES, and 0.05% (w/v) bovine serum albumin (BSA), pH 7.4] for intact respirometry. For permeabilized synaptosomes, the samples were washed once with MAS (70 mM sucrose, 220 mM mannitol, 5 mM KH_2_PO_4_, 5 mM MgCl_2_, 2 mM HEPES, 1 mM EGTA, and 0.05% fatty acid-free BSA, pH 7.2) and brought to a final volume of 150 μL per well containing 4 mM ADP, 3 nM recombinant perfringolysin O (rPFO), and either 5 mM pyruvate and 5 mM malate for complex I or 5 mM succinate and 2 μM rotenone for complex II. The microplate was incubated and loaded into the Seahorse XF96 extracellular flux analyzer (Agilent, Santa Clara, CA). During measurement in intact synaptosomes, four compounds were injected in the following order: oligomycin A (3 μM), FCCP (2 μM), and antimycin A in combination with rotenone (2 + 2 μM), all at final concentrations.

All experiments were performed at 37°C. The protocol for the measurement of oxygen consumption rate (OCR) is as previously described (56). A minimum of three technical replicates were performed in each measurement.

Synaptosome isolation and the respirometry assay were performed for 3 days from EAE day 42 to 44 in the first experiment and for 4 days from EAE day 42 to 45 in the second experiment. Each procedure day had *n* = 2 per group from each group. The absolute OCR was normalized by the controls for each day to account for day-to-day variability in OCR. A minimum of 6 mice per group per experiment were used as biological replicates for each measurement to determine statistical differences per disease.

### 2.9. Electron microscopy

In the study, 50 μg synaptosomes were fixed in 2.5% glutaraldehyde and 2% formaldehyde in 0.1 M sodium cacodylate buffer for 1 h. After wash, samples were embedded in 4% agarose gel and post-fixed in 1% osmium tetroxide. After wash, samples were dehydrated through a graded series of ethanol concentrations and propylene oxide. After infiltration with Eponate 12 resin, the samples were embedded in fresh Eponate 12 resin and polymerized at 60°C for 48 h. Ultrathin sections of 70 nm thickness were prepared and placed on former carbon-coated copper grids and stained with uranyl acetate and lead citrate. The grids were examined using a JEOL 100CX transmission electron microscope at 60 kV, and images were captured by an AMT digital camera (Advanced Microscopy Techniques Corporation, model XR611; Electron Microscopy Core Facility, UCLA Brain Research Institute).

### 2.10. SDS–PAGE and western blotting

Whole cortical lysate or synaptosome fraction were diluted in 4x Laemmli Sample Buffer (Bio-Rad, Hercules, CA, #1610747) with a supplement of DTT to a final concentration of 50 mM and were denatured by boiling for 5 min. For the detection of mitochondrial oxidative phosphorylation system (OXPHOS) proteins, instead of boiling, the samples were incubated at 37°C for 5 min. In the study, 5 μg of total protein were loaded into 4–15% Mini PROTEAN TGX precast gels (Bio-Rad, Hercules, CA, #4561084), and gel electrophoresis was performed in Mini-PROTEAN Tetra Vertical Electrophoresis Cell (Bio-Rad, Hercules, CA, #165–8004) under constant voltage at 200 V for 30 min.

Proteins were transferred to nitrocellulose membrane (pore size: 0.2um, GE Healthcare Life science, Little Chalfont, United Kingdom, #10600004) in Mini-PROTEAN^®^ Mini Trans-Blot^®^ Module (Bio-Rad, Hercules, CA, #170–3935) under 400 mA constant current for 45 min at 4°C. Blots were blocked with 1% nonfat dry milk in 1xPBST and incubated with primary antibodies diluted in 1% nonfat dry milk /PBST overnight at 4°C. The next day, blots were washed 3 × 5 min in 1xPBST, probed containing HRP-conjugated secondary antibodies (1:10,000, Thermo Fisher Scientific, Rockford, IL, #G-21234 and #31430), diluted in 1xPBST for 2 h at room temperature, and rinsed again for 3 × 5 min in 1xPBST. Detection was achieved using Clarity™ Western ECL Substrate (BioRad, Hercules, CA, #1705060) and imaged by Azure c300 (Azure Biosystems, Dublin, CA). The following primary antibodies were used: mtOXPHOS cocktail (at 1:250, for combined ATP synthase F1 subunit alpha (ATP5B), ubiquinol cytochrome c reductase core protein 2 (UQCRC2), cytochrome c oxidase I, mitochondrial (MTCO1), succinate dehydrogenase complex, subunit B (SDHB), and NADH:ubiquinone oxidoreductase subunit B8 (NDUFB8) detections, Abcam, Waltham, MA, cat#ab110413), mouse anti-β-actin (at 1:3000, Cell Signaling Technology, Danvers, MA, cat#3700), mouse anti-postsynaptic density protein 95 (PSD95; at1:1000, BioLegend, San Diego, CA, cat#810301), mouse anti-glutamate receptor 2 (GLUR2; at 1:100, Biolegend, cat#810501), rabbit anti-GST-pi (at 1:500, Enzo Life Sciences, Farmingdale, NY, cat#ADI-MSA-102), and rabbit anti-voltage-dependent anion channel ½ (VDAC1/2; at 1:500, Proteintech, Rosemont, IL, cat# 10866-1-AP).

### 2.11. Statistical analysis

Mice were randomly assigned to experimental groups. Investigators were blinded to group allocation during data collection and analysis. Points represent individual animals. For all box-and-whisker plots, the center represents the median, the boxes represent the interquartile range, and the whiskers represent the highest and lowest values excluding outliers. Prism 9 (GraphPad) was used for all histological and respirometry data analysis. The statistical difference between groups was determined using the Mann–Whitney *U*-test. Statistical significance was reported as a *p < 0.05; **p < 0.01; *** *p* < 0.001; and ****p < 0.0001.

### 2.12. Data availability

Datasets generated in this study are available in the GEO (Gene Expression Omnibus)^5^ under the accession number GSE239455.

## 3. Results

### 3.1. Decreased neurons in the cerebral cortex in male mice with chronic experimental autoimmune encephalomyelitis and sex differences in the transcriptome of cortical neurons

**Figure 1:**
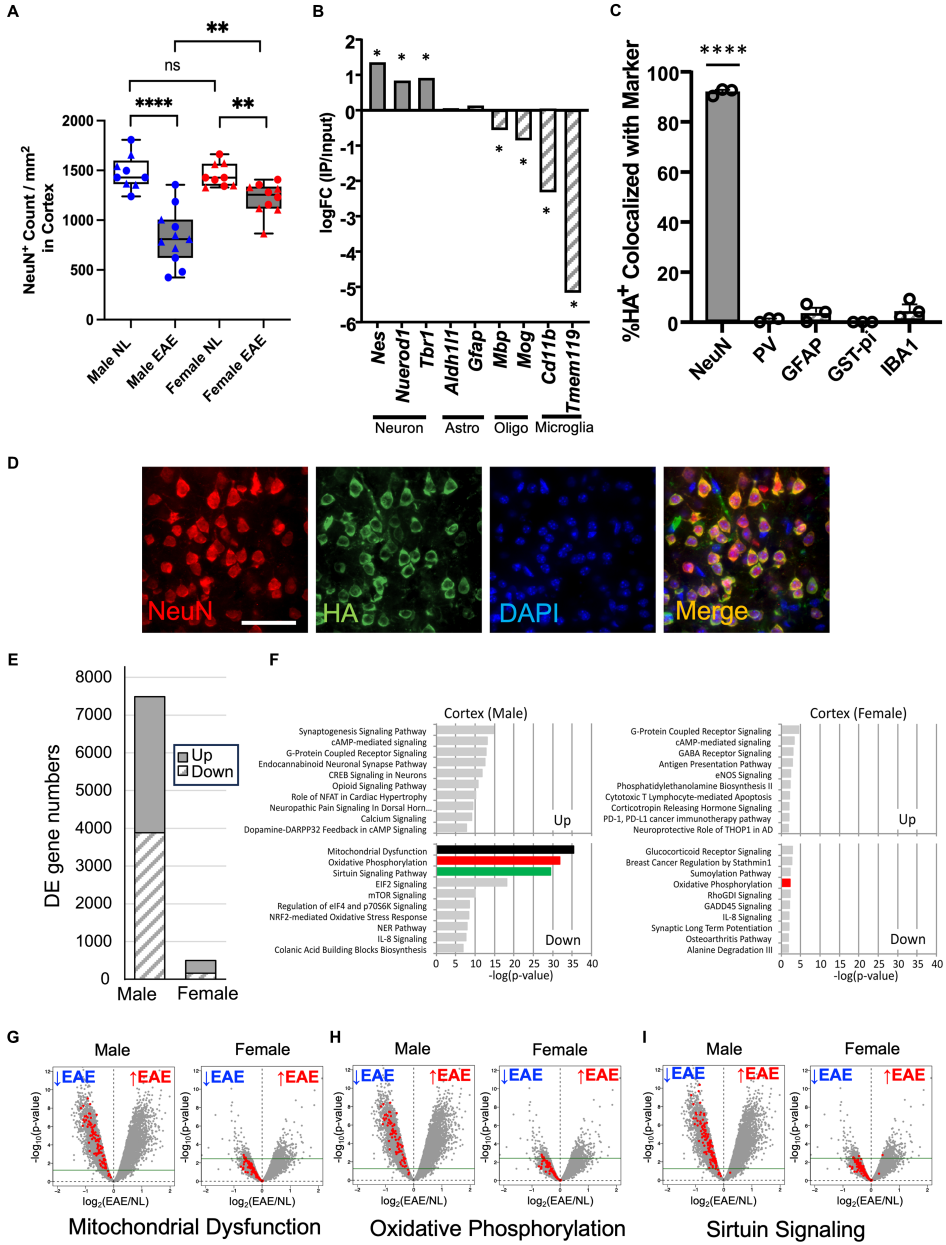
Worse neuronal loss in the cerebral cortex in male mice with chronic EAE and sex differences in the neuron-specific transcriptome. **(A)** Quantification of NeuN^+^ cortical neurons from healthy age- and sex-matched controls (NL, white) and EAE (EAE, gray), with male mice represented in blue and female mice in red. Data from two separate experiments (circle: 1st experiment, triangle: 2nd experiment). EAE showed a significant decrease of NeuN^+^ number as compared to control in male (*p* < 0.0001) and female mice (*p* = 0.0014). Additionally, male mice showed a significant decrease in NeuN^+^ number compared to female mice in EAE (*p* = 0.0028) but not in controls (*p* = 0.5919; *n* = 11 mice in EAE, *n* = 9 mice in control; **** *p* < 0.0001, **p < 0.01). *p*-values were calculated by the Mann–Whitney *U*-test. **(B)** RNA enrichment analysis. In immunoprecipitated RNA samples from FIGURE 1 (Continued)NSE-Cre:RiboTag mice, neuronal markers (*Nestin*, *NeuroD1*, and *Tbr1*) were enriched, while astrocytes (*Aldhl1l* and *Gfap*), oligodendrocytes (*Mbp* and *Mog*), and microglia genes (*Cd11b* and *Tmem119*) showed no enrichment. Asterisks: FDR < 0.1 (edgeR). **(C)** Quantification of HA co-localization with NeuN^+^ (neurons), PV^+^ (interneurons), GFAP^+^ (astrocytes), GST-pi^+^ (oligodendrocytes), and IBA1^+^ cells (microglia/macrophages). *n* = 3, error bars represent SEM. **** *p* < 0.0001. *p*-values were calculated by the one-sample *t*-test. **(D)** Representative 40× images of NeuN (red), HA (green), and DAPI (blue) in a merged form showing co-localization (yellow), all from the cerebral cortex of NSE-Cre:RiboTag mice. Bar = 40um. **(E)** Differentially expressed gene (DE gene) numbers comparing EAE male mice with control male mice and EAE female mice with control female mice. FDR < 0.1, logFC<−0.25 or 0.25 < logFC. EAE *n* = 5 (male) or *n* = 4 (female) and control *n* = 4 (male) or *n* = 4 (female). **(F)** Top 10 canonical pathways for upregulated and downregulated genes in male EAE mice compared to male control mice (left) and female EAE mice compared to female control mice (right; FDR < 0.1, logFC<−0.25 or 0.25 < logFC). Downregulated genes in EAE male mice were enriched in mitochondrial dysfunction (black), oxidative phosphorylation (red), and sirtuin signaling (green) pathways. Pathways are shown in a descending order of significance. **(G–I)** Volcano plots to visualize the genes in mitochondrial dysfunction **(G)**, oxidative phosphorylation **(H)**, and sirtuin signaling **(I)** pathways, within each volcano panel male EAE mice compared to male control mice (left) and female EAE mice compared to female control mice (right). Green line: FDR = 0.1.

A decrease in neurons in cortical layer V of the cerebral cortex during chronic EAE has been described in a previous study (36). Whether there is a sex difference in this loss of cortical neurons during chronic EAE remains unknown. Here, immunostaining of neurons (NeuN^+^) was done on cerebral cortex tissues from male and female mice with chronic EAE (1st experiment EAE day 47; 2nd experiment EAE day 44) and from healthy age- and sex-matched controls. As expected, there was no sex difference in EAE walking scores in the C57BL/6 model (Supplementary Figure 1). The average day of onset was a mean of 15.25 in the 1st experiment (15.63 in female mice; 14.83 in male mice) and 14.47 in the 2nd experiment (14.90 in female mice; 13.86 in male mice), maximum scores (scale of 0 to 5) were 4.0 in the 1st experiment (4.0 in female mice; 4.0 in male mice) and 4.5 in the 2nd experiment (4.5 in female mice; 4.5 in male mice), and average scores over all days were 2.09 in the 1st experiment (1.98 in female mice; 2.20 in male mice) and 2.18 in the second experiment (2.04 in female mice; 2.39 in male mice). Despite no sex difference in standard EAE walking scores, the degree of cortical neuron loss in male mice with EAE compared to healthy male mice was more pronounced than in female mice with EAE compared to healthy female mice (Figure 1A; Supplementary Figure 2).

Changes in gene expression in cortical neurons during chronic EAE in each sex were then investigated using RiboTag technology (8, 43). NSE-Cre mice were crossed with RiboTag mice (NSE-Cre:RiboTag) to generate mice expressing HA-tagged ribosomal protein specifically in neurons. Cerebral cortex tissues were acquired, and neuron-specific RNAs were isolated. The specificity of HA labeling in NSE-Cre:RiboTag mice was confirmed through RNA-seq analysis (Figure 1B) and immunostaining (Figures 1C,D). At the RNA level, neuronal genes (*Nestin*, *NeuroD1*, and *Tbr1*) were enriched in the immunoprecipitated neuronal RNA, whereas astrocytes (*Aldhl1l* and *Gfap*), oligodendrocytes (*Mbp* and *Mog*), and microglia genes (*Cd11b* and *Tmem119*) were not enriched (Figure 1B). At the protein level, HA staining co-localized with the neuron marker (NeuN), while there was no significant co-localization with other CNS cell markers: parvalbumin-expressing interneurons (PV), astrocytes (GFAP), oligodendrocytes (GST-pi), or microglia/macrophages (IBA1; Figures 1C,D). These results validated the neuron-specificity of RNAs isolated from the cerebral cortex of NSE-Cre:RiboTag mice.

Genome-wide transcriptome analyses were then carried out on neuron-specific RNAs from male and female NSE-Cre: RiboTag mice with chronic EAE, as well as from age- and sex-matched healthy controls. In male mice with EAE as compared to male controls, 7,487 genes were differentially expressed (upregulated 3,601, downregulated 3,886), while female mice with EAE compared to female controls showed only 495 genes differentially expressed (upregulated 336, downregulated 159; FDR < 0.1, logFC<−0.25 or 0.25 < logFC; Figure 1E). A canonical pathway analysis (Ingenuity Pathway Analysis)^6^ revealed that the top three pathways showing the most enrichment in male mice during EAE were the mitochondrial dysfunction, oxidative phosphorylation, and sirtuin signaling pathways, with each downregulated (Figure 1F). In contrast, female mice showed less significant enrichment of pathways during EAE. Male-specific downregulation of mitochondria-related genes was further confirmed in the additional gene set enrichment analysis using GO (Gene Ontology) database, while female mice showed almost no significant enrichment (Supplementary Figure 3). Volcano plots showing the expression of genes in each of the top three pathways most enriched in male mice are shown for both sexes (see Figures 1G–I). In contrast to EAE male mice, the majority of pathway genes in EAE female mice were below the threshold (FDR = 0.1) and not significantly different when comparing EAE to the sex-matched healthy control.

Together, transcriptome changes in cortical neurons during chronic EAE were robust in male mice and minimal in female mice, and differences in expression of mitochondria-related genes were primarily responsible for this sex difference.

### 3.2. Mitochondrial morphology in neurons of the cerebral cortex is abnormal in male mice with experimental autoimmune encephalomyelitis

Mitochondrial fusion and fission play a vital role in maintaining mitochondrial homeostasis. Our transcriptome analyses of neurons in the cerebral cortex during EAE revealed dysregulation in the expression of genes involved in fusion and fission (*Fis1*, *Dnm1l*, *Mfn1*, and *Opa1*) in male mice but not female mice (Supplementary Table 1).

**Figure 2:**
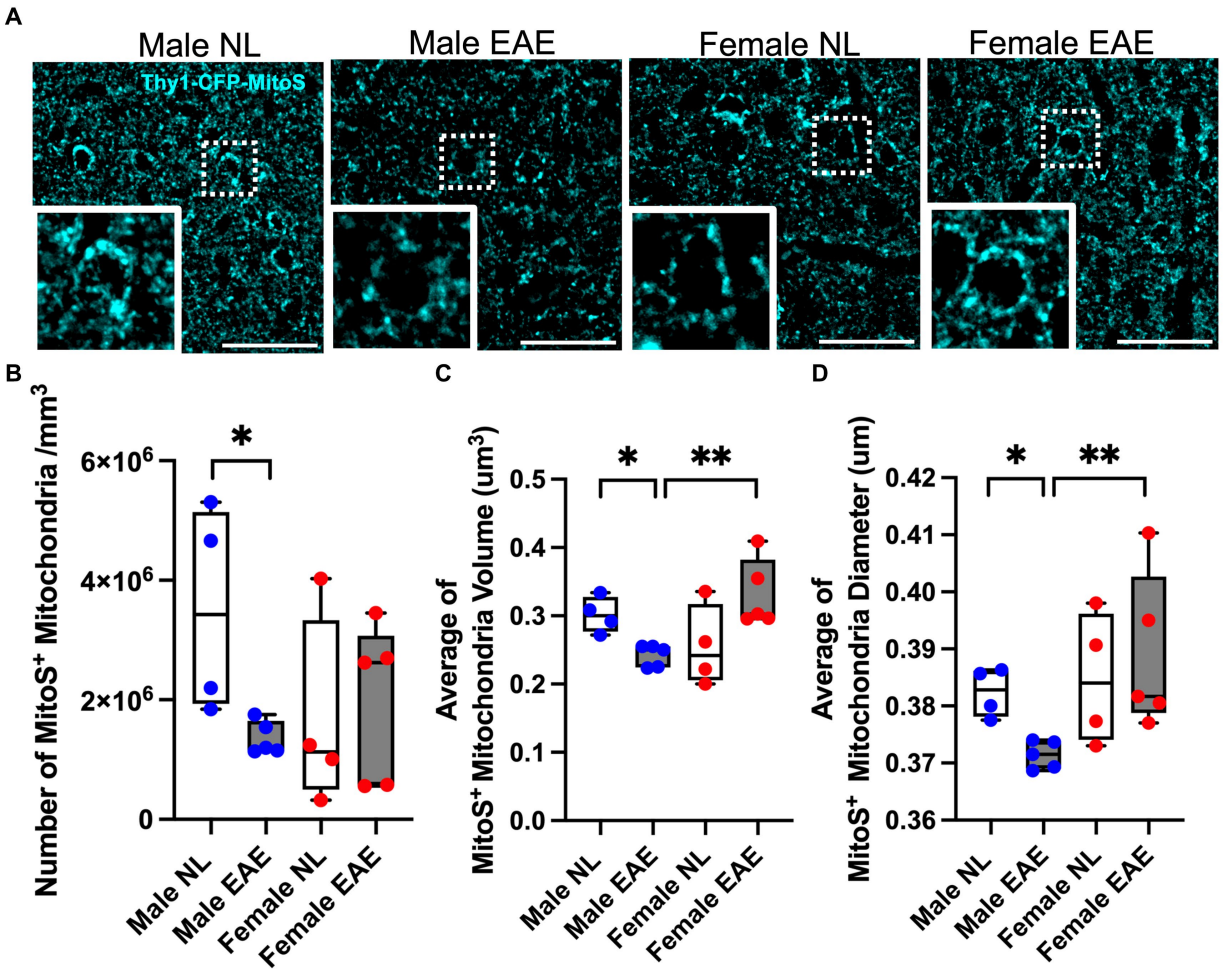
Mitochondria morphology in the cerebral cortex. **(A)** Representative Thy1- CFP-MitoS^+^ (MitoS^+^) images in the cerebral cortex of male control (Male NL) and EAE (Male EAE) mice; female control (Female NL) and EAE (Female EAE) mice. Bar = 40 μm. Inset: Magnification at 100×. **(B–D)** Morphometric quantification of the MitoS^+^ mitochondrial number **(B)**, volume **(C)**, and diameter **(D)** from control (NL, white) and EAE (EAE, gray); male mice (blue) and female mice (red). EAE male mice showed a significant decrease in MitoS^+^ number (*p* = 0.0159, B), volume (*p* = 0.0159, C), and diameter (*p* = 0.0159; D) as compared to control male mice. *n* = 4–5 per group. MitoS^+^ mitochondrial volume (*p* = 0.0079; C) and diameter (*p* = 0.0079; D) were significantly decreased in EAE male mice compared to EAE female mice (*p < 0.05; **p < 0.01). *p* values were calculated by the Mann–Whitney *U*-test.

Examination of the morphology of mitochondria in cortical neurons was then done using Thy1-CFP-MitoS mice (Figure 2A). EAE male mice showed a significant reduction in the numbers of cortical MitoS^+^ mitochondria compared to normal control male mice (Figure 2B). Moreover, MitoS^+^ morphology in EAE male mice exhibited a significant decrease in volume (Figure 2C) and diameter (Figure 2D). In contrast, no significant differences in cortical MitoS^+^ number, volume, or diameter were observed in EAE female mice compared to normal control female mice. Notably, a significant decrease in cortical MitoS^+^ mitochondria volume and diameter was also observed when comparing EAE male to EAE female mice (Figures 2C,D). This demonstrated that neuron-specific mitochondrial morphology was affected in the cerebral cortex of EAE male mice more than EAE female mice.

### 3.3. Mitochondrial respiration in cortical synaptosomes is impaired in males with experimental autoimmune encephalomyelitis

Since cortical neurons showed changes in gene expression in the mitochondrial and oxidative pathways in EAE male mice, we next investigated mitochondrial function in EAE and healthy control male and female mice. To determine mitochondrial function in cortical neurons, we isolated synaptosomes since they are derived from the termini of neurons and contain mitochondria. The isolation of synaptosomes was verified using electron microscopy to confirm the presence of membrane-enclosed structures housing mitochondria (Supplementary Figure 4B) and by western blotting to confirm the enrichment of synaptic markers and the presence of mitochondrial proteins (Supplementary Figure 4C). Following this validation, isolated synaptosomes underwent functional respirometry assays.

**Figure 3:**
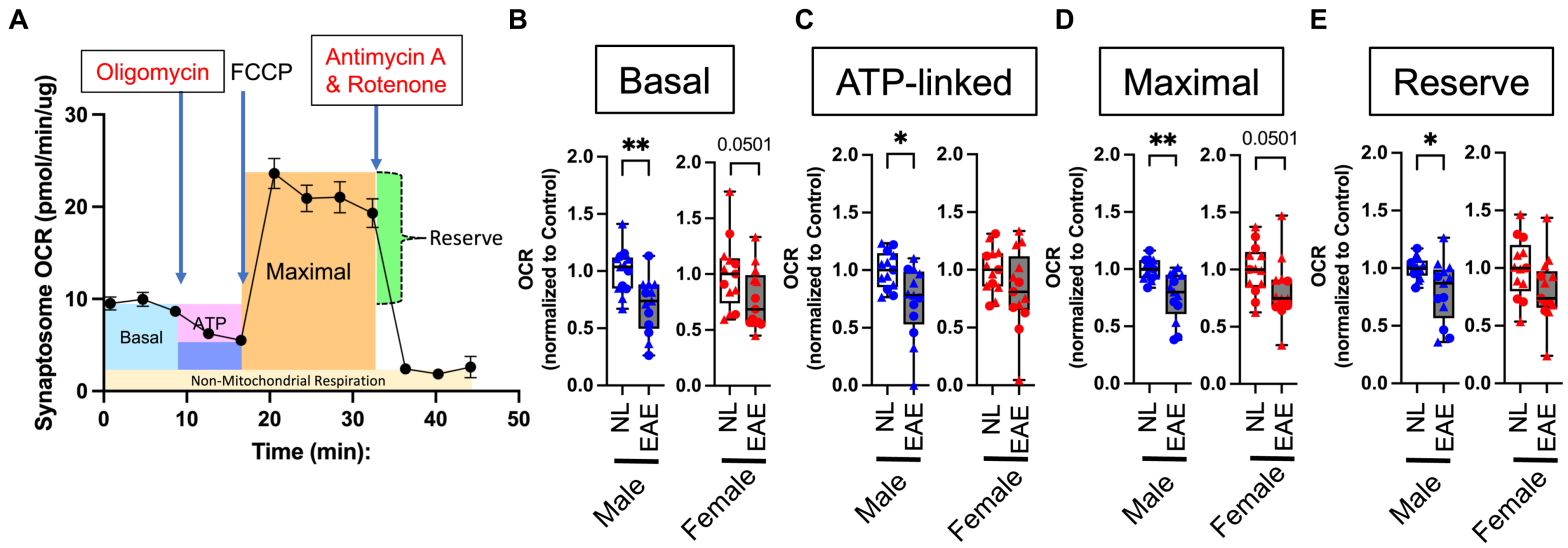
Intact synaptosome oxygen consumption. **(A)** Representative mitochondrial respiratory profile from intact synaptosomes. The figure shows across time (in min) basal OCR (blue); OCR after inhibition of ATP synthase through the addition of oligomycin (pink); maximal stimulatory capacity of synaptosomes in response to the uncoupling agent FCCP (orange); reserve (calculated from maximal OCR minus basal OCR, green); and non-mitochondrial respiration (measured after combined treatment with rotenone and antimycin A to completely inhibit mitochondrial respiration, yellow). **(B–E)** Quantitative analysis of mitochondrial OCR in EAE mice compared to normal healthy control mice for basal **(B)**, ATP-linked **(C)**, maximal **(D)**, and reserve **(E)** parameters using intact cortical synaptosomes from control (NL, white) and EAE (EAE, gray); male mice (blue) and female mice (red). Data shown are from two separate experiments (circle symbol: 1st experiment, triangle symbol: 2nd experiment). EAE male mice showed a significant decrease in basal OCR (*p* = 0.0061; B), ATP-linked (*p* = 0.014; C), maximal (*p* = 0.0012; D), and reserve (*p* = 0.0191; E) parameters, each as compared to NL control male mice. In contrast, EAE female mice compared to NL female mice showed a trend of decrease in two of four parameters: basal OCR (*p* = 0.0501; B) and maximal OCR (*p* = 0.0501; D) with no significant differences in any parameters. OCR in EAE mice normalized by that NL control. *n* = 14 per group. * *p* < 0.05; ** *p* < 0.01. *p* values were calculated by the Mann–Whitney *U*-test.

A schematic of a mitochondrial respirometry profile from intact synaptosomes is shown in Figure 3A. Intact synaptosomes derived from EAE male mice compared to normal control male mice showed a significant reduction of oxygen consumption rate (OCR) in basal (Figure 3B), ATP-linked (Figure 3C), maximal (Figure 3D), and reserve (Figure 3E) parameters. In contrast, EAE female mice compared to control female mice only showed a trend of decrease in two of the four parameters: basal OCR (Figure 3B) and maximal OCR (Figure 3D).

**Figure 4:**
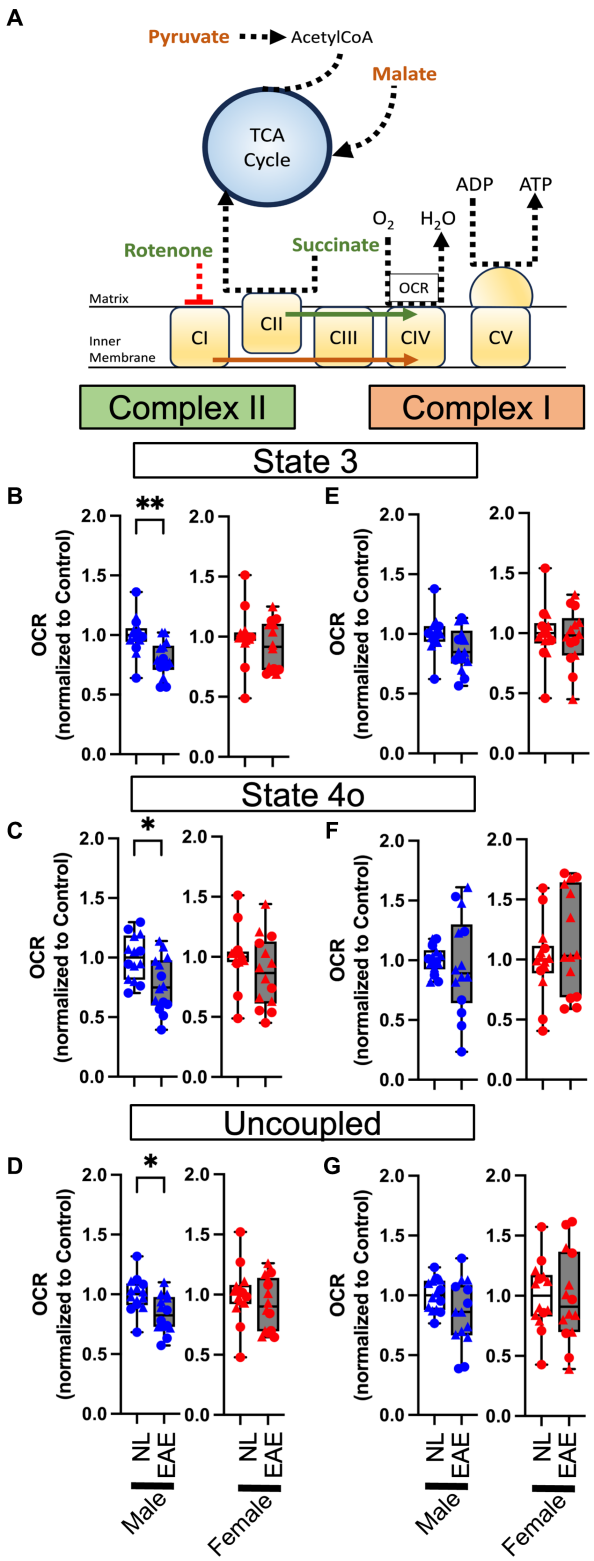
Permeabilized synaptosome oxygen consumption. **(A)** Oxidation phosphorylation diagram for complex I- (orange) and complex II- (green) mediated respirometry. Permeabilized synaptosomes were treated with two different substrate and/or inhibitor combinations: pyruvate in combination with malate for complex I-mediated respirometry or with succinate with rotenone for complex II-mediated respiration. Permeabilized synaptosomes were stimulated with ADP to induce coupled respiration (State 3), followed by oligomycin to induce proton-leak respiration (State 4o), as well as with the mitochondrial uncoupler FCCP to reveal maximal uncoupled respiration (uncoupled). **(B–G)** Quantitative analysis of mitochondrial OCR in EAE mice compared to NL control for state 3 **(B,E)**, state 4o **(C,F)**, and uncoupled **(D,G)** parameters in complexFIGURE 4 (Continued)II-mediated **(B–D)** and complex I-mediated **(E–G)** respirometry using permeabilized cortical synaptosomes from control (NL, white) and EAE (EAE, gray); male mice (blue) and female mice (red). Data shown are from two separate experiments (circle symbol: 1st experiment, triangle symbol: 2nd experiment). EAE male mice showed a significant decrease in OCR complex II-mediated respiration for state 3 (*p* = 0.0016, K), state 4o (*p* = 0.0141, L), and uncoupled (*p* = 0.00102, M) parameters, each as compared to NL control male mice. In contrast, OCR in EAE female mice was intact in complex II-mediated respiration. There were no differences in any complex I-mediated respiration parameters in EAE compared to NL in either sex. *n* = 14 per group. * *p* < 0.05; ** *p* < 0.01. *p* values were calculated by the Mann–Whitney *U*-test.

Next, we conducted complex-specific respiration assays using permeabilized synaptosomes, using a combination of either pyruvate and malate (substrates for complex I) or succinate (substrate for complex II) and rotenone (complex I inhibitor; see a diagram in Figure 4A). There were no differences in any complex I-mediated respiration parameters in EAE compared to control in either sex (Figures 4E–G). However, there were effects of EAE on complex II-mediated respiration parameters, and this revealed a sex difference. EAE male mice showed a significant decrease in OCR complex II-mediated respiration for state 3 (Figure 4B), state 4o (Figure 4C), and uncoupled (Figure 4D) parameters each as compared to control male mice. In contrast, OCR in EAE female mice was not decreased for complex II-mediated respiration.

Finally, we compared functional respiration parameters in healthy control male and female mice to ascertain if absolute OCR differences might exist independent of disease. This was not the case, since no significant OCR differences were observed between male and female mice in the control groups across basal, ATP-linked, maximal, and reserve parameters in intact synaptosomes (Supplementary Figure 5). Additionally, there were no differences in complex II- or I-mediated respiration assays for state 3, state 4o, and uncoupled parameters in permeabilized synaptosomes (Supplementary Figure 6).

Mitochondria respirometry assays demonstrated that EAE induces impairment of mitochondrial respiration in cortical synaptosomes, which was worse in male mice than in female mice, particularly in complex II-mediated respiration.

## 4. Discussion

Neuronal transcriptome, mitochondrial morphology, and functional respirometry assays in synaptosomes revealed worse outcomes in the cerebral cortex of male EAE mice than in female EAE mice of the C57BL/6 strain. This is consistent with worse neurodegeneration in MS men and reveals a model and a target to develop treatments with the potential to prevent cortical pathology and mitigate disability progression in MS men.

In addition to considering translation of our preclinical findings to MS, one can also discuss possible mechanisms leading to our preclinical observations. Findings that male mice have more significant mitochondrial and oxidative gene expression changes in cortical neurons, abnormal mitochondrial morphology, and decreased oxygen consumption rates in synaptosomes could be driven by an effect of sex chromosomes, sex hormones, or both. Sex chromosomes and sex hormones can act in a synergistic or antagonistic manner in a given process (57). Compensatory mechanisms may have arisen during evolution to promote survival of each sex, reaching an optimal balance between sex chromosome and sex hormone influences. How each sex reaches this balance is likely distinct given the differences in sex hormones (estrogen vs. testosterone) and sex chromosomes (XX vs. XY) (58).

With regard to autoimmunity and neuroprotection, we hypothesize that XX is proinflammatory and estrogen is neuroprotective. This is consistent with female mice developing more robust immune responses to infections and being predisposed to autoimmunity but having more resilience to brain injury. It follows that male mice would have less robust immune responses to infections, be less susceptible to autoimmune diseases, and exhibit more neurodegeneration during brain injury. Evidence supporting this hypothesis is supported by the use of the four core genotype model (59). The XX genotype was shown to be more proinflammatory in the relapsing remitting EAE model in the SJL genetic background, in the chemically induced lupus model in the SJL, and spontaneous lupus in the NZM.2328 genetic background (60, 61). Conversely, the XY genotype was shown to confer worse neurodegeneration in the chronic EAE model on the C57BL/6 background (62) and in the human amyloid precursor protein model of Alzheimer’s disease (63). Notably, the proinflammatory effects of XX in active EAE in SJL mice were verified by using adoptive transfers of immune cells that were XX or XY into a common recipient (61). More neurodegenerative effects of XY compared to XX in active EAE in C57BL/6 mice were shown using bone marrow chimeras to ensure the same immune system with the central nervous system that differed in sex chromosomes (62). Findings here where the neuronal transcriptome, mitochondrial morphology, and functional respirometry assays in synaptosomes demonstrated more abnormalities in the cerebral cortex of male C57BL/6 mice with chronic EAE are consistent with a more neurodegenerative effect of XY using this same chronic EAE model (62). Limitations to this interpretation include the following: Bone marrow chimeras were not used here, so male immune cells may have contributed to worse neuronal outcomes in the cerebral cortex of males (64). Findings here could theoretically be due to testosterone in male mice causing worse neurodegeneration or estrogen in female mice being neuroprotective. These are not mutually exclusive. Repeating experiments in gonadectomized mice to remove activational effects of sex hormones would be informative. There could also be organizational effects of sex hormones, e.g., an effect of differences in testosterone vs. estrogen during development, which cannot be addressed merely by gonadectomy at adult ages. To investigate organizational effects of sex hormones, experiments using the four core genotype mice (59) are needed. Comparison would be made between XY mice with ovaries vs. XY-*Sry* mice with testes, where developmental hormones differ, and sex chromosome complement is the same. These are interesting future directions to discern mechanisms underlying sex differences observed here in cortical neurons during chronic EAE.

Further discussion is warranted regarding the role of sex hormones on molecular outcomes observed here. An effect of sex chromosome complement is not mutually exclusive of a contribution of circulating testosterone or estradiol in gonadally intact male and female adult mice. Indeed, both testosterone and estradiol have neuroprotective properties in MS models. Specifically, testosterone has been shown to bind androgen receptors to induce remyelination and can also be protective via its conversion to estradiol by aromatase in the brain (65–67). Estradiol mediates neuroprotection by activating estrogen receptors alpha (ERα) and/or beta (ERβ) in the central nervous system of EAE mice. ERα has been shown to act on astrocytes to confer protection by modifying immune cell recruitment (45, 68, 69), while ERβ acts on oligodendrocytes and microglia to induce remyelination (70–72). In conditions other than MS or EAE, ERα and ERβ expression was demonstrated in mitochondria, and ligation of estrogen receptors conferred protection in mitochondrial function within neurons as well as other cells and tissues (73–76).

In humans, the loss of either testosterone with andropause or estradiol with menopause is believed to be deleterious for neurogenerative changes in MS, brain aging, and Alzheimer’s disease, as reviewed in Voskuhl and Itoh (18). The difference in timing of andropause and menopause may play a role in men having worse disability progression and more gray matter substructure atrophy than women. Data for these findings were generally derived from MS patients aged 18–60 years, with the mean age of early 40s. Testosterone declines in men by 1–2% per year starting at the age of 30 years. Meanwhile, women in their early 40s maintain the levels of estradiol since menopause occurs at a mean age of 52 years. Indeed, when women undergo menopause and have loss of estradiol, it is associated with disability worsening (77–83). Thus, worse disability progression in men compared to women with MS may be age-dependent, e.g., an age when men exhibit effects of andropause but before women undergo menopause (18).

In summary, the ultimate effect of biologic sex on adults with MS is due to changes in sex hormones during aging in the setting of inheritance of a given sex chromosome complement. Sex hormones and sex chromosomes can have opposing effects on disease, in part through differential effects on the immune system vs. the CNS. Even when there is no overall sex difference in a clinical outcome, there could be very important disease-modifying effects of sex hormones or sex chromosomes that reach equivalence regarding the net impact on disease. This is the rationale for studying the effects of sex hormones and sex chromosomes not only in diseases where there is a sex difference but also in diseases not characterized by an overall sex difference. It can lead to the discovery of naturally existing disease modifiers in each sex.

## Data availability statement

Datasets generated in this study are available in the GEO (Gene Expression Omnibus; www.ncbi.nlm.nih.gov/geo/) under the accession number GSE239455.

## Ethics statement

All animal procedures were done in accordance to the guidelines of the National Institutes of Health and the Chancellor’s Animal Research Committee of the University of California, Los Angeles Office for the Protection of Research Subjects. The study was conducted in accordance with the local legislation and institutional requirements.

## Author contributions

NI: Writing – review & editing, Formal analysis, Investigation, Methodology. YI: Formal analysis, Investigation, Methodology, Writing – review & editing. LS: Formal analysis, Investigation, Methodology, Writing – review & editing. RV: Writing – review & editing, Conceptualization, Funding acquisition, Writing – original draft.

## Figures and Tables

**Table 1:** 

EAE	Experimental autoimmune encephalomyelitis
NeuN	Neuronal Nuclear Protein
NSE	Neuron Specific Enolase
HA	Hemagglutinin
RNA	Ribonucleic acid
RNA-seq	RNA sequencing
*Nes*	Nestin
*NeuroD1*	Neurogenic differentiation 1
*Tbr1*	T-Box Brain Transcription Factor 1
*Aldh1l1*	Aldehyde Dehydrogenase 1 Family Member L1
*Gfap*	Glial fibrillary acidic protein
*Mbp*	Myelin basic protein
*Mog*	Myelin oligodendrocyte glycoprotein
*Cd11b*	CD11 Antigen-Like Family Member B
*Tmem119*	Transmembrane protein 119
PV	Parvalbumin
CNS	Central nervous system
GST-pi	Glutathione S-transferase pi
IBA1	Ionized calcium-binding adapter molecule 1
FDR	False Discovery Rate
Log FC	Log-fold change
*Fis1*	Fission, mitochondrial 1
*Dnm1l*	Dynamin 1-like
*Mfn1*	Mitofusin 1
*Opa1*	OPA1, mitochondrial dynamin like GTPase
Thy1	Thymus cell antigen 1, theta
CFP	Cyan Fluorescent Protein
OCR	Oxygen consumption rate
ATP	Adenosine triphosphate
FCCP	Carbonyl cyanide 4-(trifluoromethoxy)phenylhydrazone
ADP	Adenosine diphosphate
PBS	Phosphate-buffered saline
PBST	Phosphate-buffered saline with Tween-20
SDS-PAGE	Sodium dodecyl-sulfate polyacrylamide gel electrophoresis
PSD95	Postsynaptic density protein 95
GLUR2	Glutamate ionotropic receptor AMPA type subunit 2
ATP5A	ATP synthase F1 subunit alpha
UQCRC2	Ubiquinol cytochrome c reductase core protein 2
MTCO1	Cytochrome c oxidase I, mitochondrial
SDHB	Succinate dehydrogenase complex, subunit B, iron sulfur (Ip)
NDUFB8	NADH:ubiquinone oxidoreductase subunit B8
VDAC1/2	Voltage-dependent anion channel 1/2

## Footnotes

^1^http://www.R-project.org/

^2^http://www.bioinformatics.babraham.ac.uk/projects/fastqc/

^3^www.qiagen.com/ingenuity

^4^https://github.com/AhsenChaudhry/Mitochondria-Analyzer

^5^www.ncbi.nlm.nih.gov/geo/

^6^www.qiagen.com/ingenuity
