# Supplementary material for: Sex differences in the neuronal transcriptome and synaptic mitochondrial function in the cerebral cortex of a multiple sclerosis model
Source: Front Neurol. 2023 Nov 2;14:1268411. doi: 10.3389/fneur.2023.1268411 (PMC10654219; doi:10.3389/fneur.2023.1268411)
Supplement: Supplementary file 1 [file Data_Sheet_1.pdf]

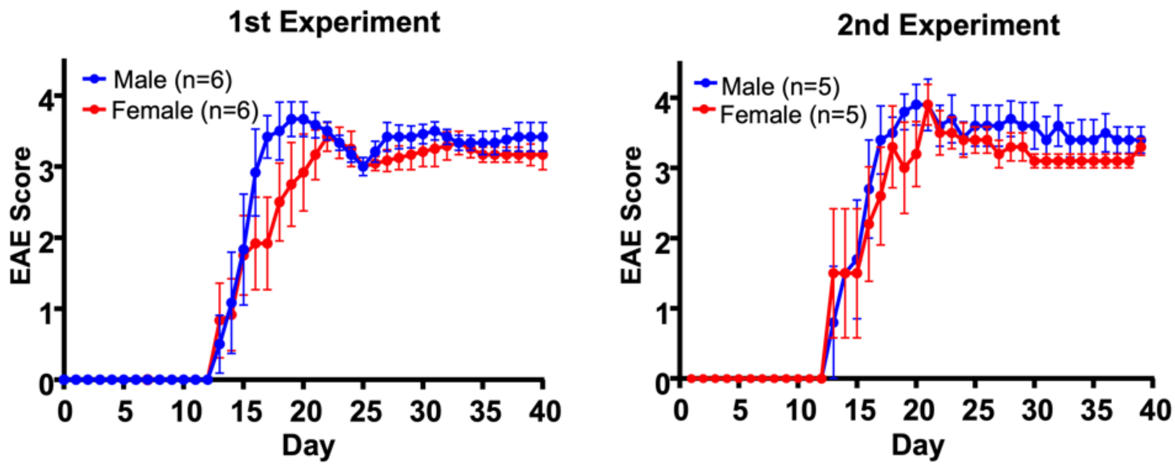


**Supplemental Figure 1. No sex difference in standard EAE walking scores.** EAE clinical score graphs of males (bule) and females (red) in 1st (left) and 2nd (right) EAE experiments. No clinical score differences between males and females were observed. 1st experiment: n=6 per group. 2nd experiment: n=5 per group. Error bars represent SEM.

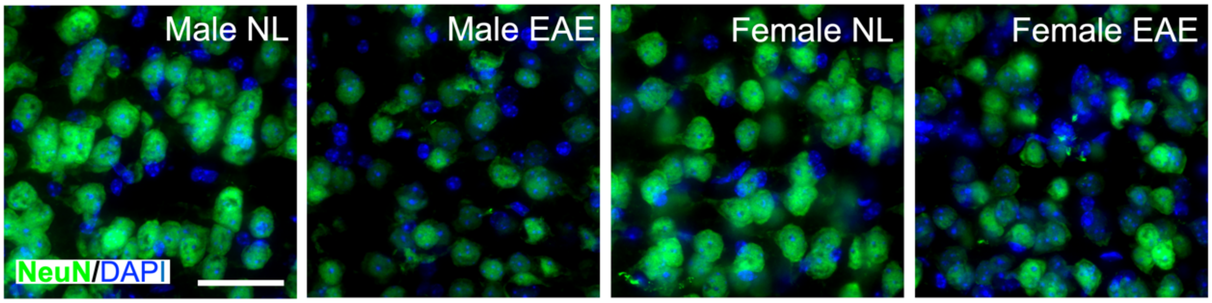


**Supplemental Figure 2. Cortical neuron loss during EAE is worse in males than in females.** Representative 40X images of NeuN$^+$ (green) cortical neurons from male control (Male NL), male EAE (Male EAE), female control (Female NL), and female EAE (Female EAE). Quantification of NeuN$^+$ cortical neurons indicated in Figure 1A. Nuclei were counterstained with DAPI (blue). Bar=40µm.

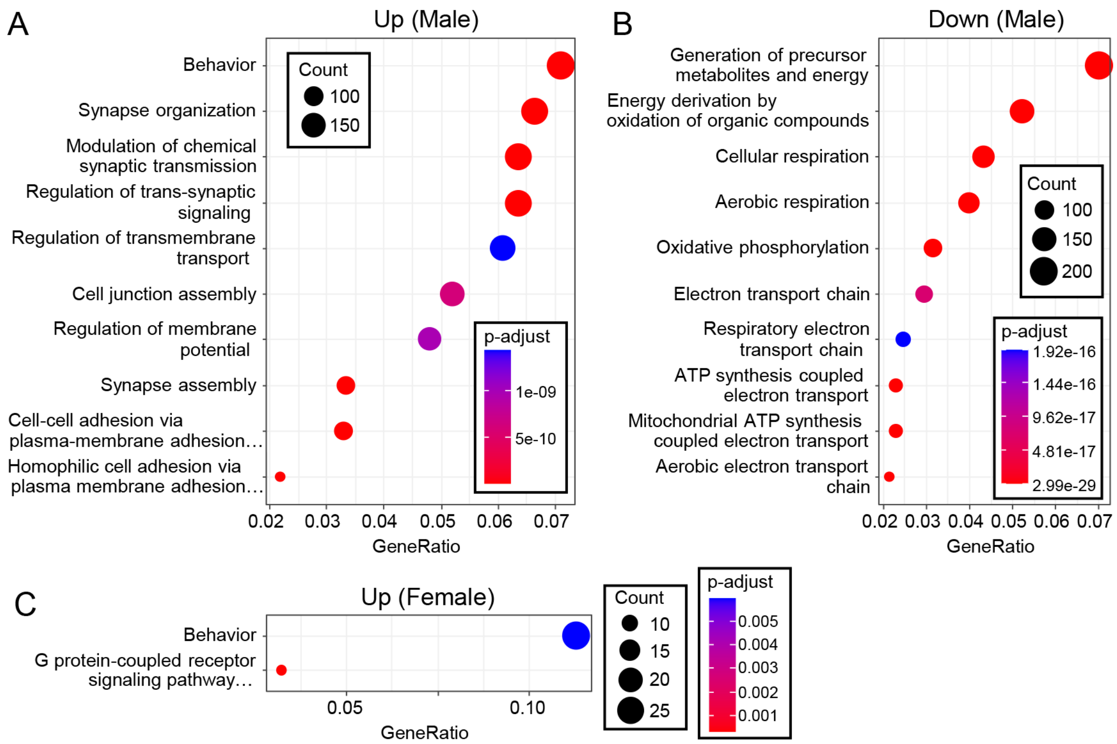


**Supplemental Figure 3. Genes downregulated in males with EAE were enriched in mitochondria-related gene ontology categories.** GO (Gene Ontology) enrichment analyses of differentially expressed genes (FDR < 0.1, logCPM > 1) were performed for **(A)** male upregulated (logFC > 0.25), **(B)** male downregulated (logFC < -0.25), and **(C)** female upregulated (logFC > 0.25) genes, each in EAE as compared with healthy controls. For female downregulated genes, there was no significant enrichment observed. Genes downregulated in male EAE were enriched in energy production, respiration, and oxidative phosphorylation **(B)**.

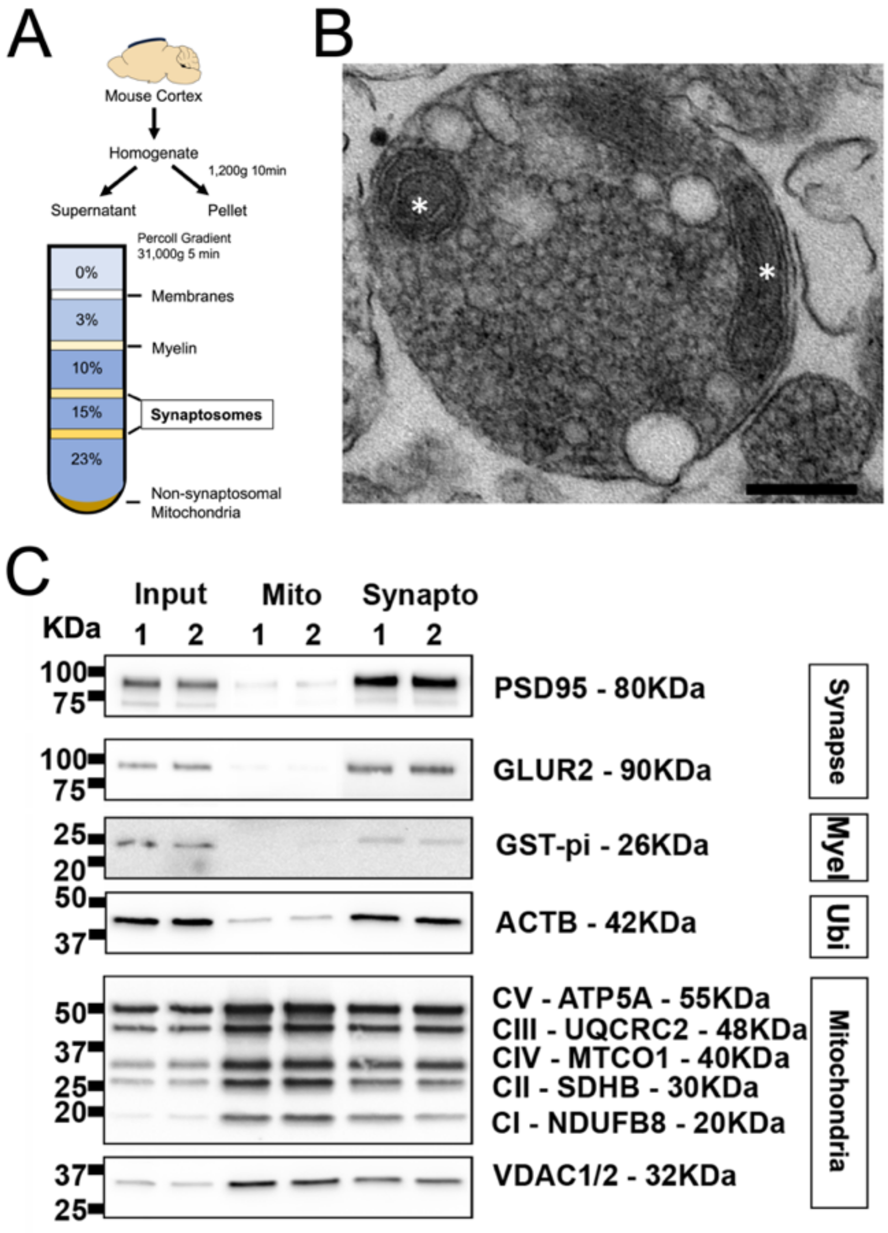


**Supplemental Figure 4. Synaptosomes quality assessment. (A)** Diagram of synaptosome isolation. **(B)** Synaptosome presence was confirmed by electron microscopy. White asterisks indicate mitochondria within enclosed membrane structures. Scale bar: 200 nm. **(C)** Equal amounts of total protein (5 μg) from the homogenate (Input), non-synaptosomal mitochondria (Mito), and synaptosomes (Synapto) were separated by SDS-PAGE and analyzed by western blot for the presence of various markers. Synaptic markers (Synapse) PSD95 and GLUR2, a myelin marker (Myel) GST-pi, a ubiquitous marker (Ubi) β-actin, and mitochondrial markers (Mitochondria) ATP5A, UQCRC2, MTCO1, SDHB, NDUFB8, and VDAC1/2 were examined. Isolated synaptosomes (Synapto) confirmed the presence of synaptic markers, PSD95 and GLUR2, as well as mitochondrial markers ATP5A, UQCRC2, MTCO1, SDHB, NDUFB8, and VDAC1/2, along with β-actin, and a decrease of GST-pi levels. In contrast, non-synaptosomal mitochondria (Mito) showed enrichment of mitochondrial markers and a decrease in synaptic markers and β-actin levels.

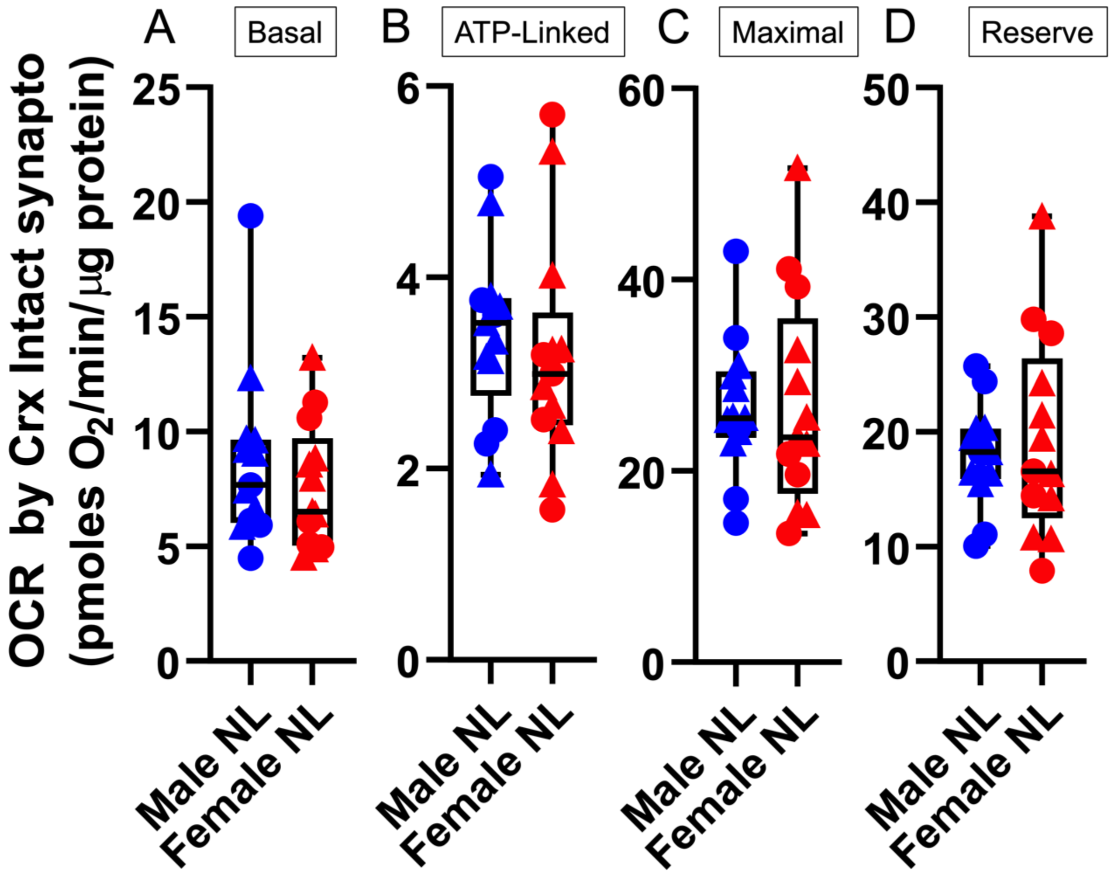


**Supplemental Figure 5. No sex differences in absolute oxygen consumption in cortical intact synaptosomes derived from healthy control males and females. (A – D)** Quantitative analysis of mitochondrial OCR for Basal (A), ATP-linked (B), Maximal (C), and Reserve (D) parameters using intact cortical synaptosomes from control (NL); male mice (blue) and female mice (red). Data shown from two separate experiments (circle symbol: 1st experiment, triangle symbol: 2nd experiment). n=14 per group.

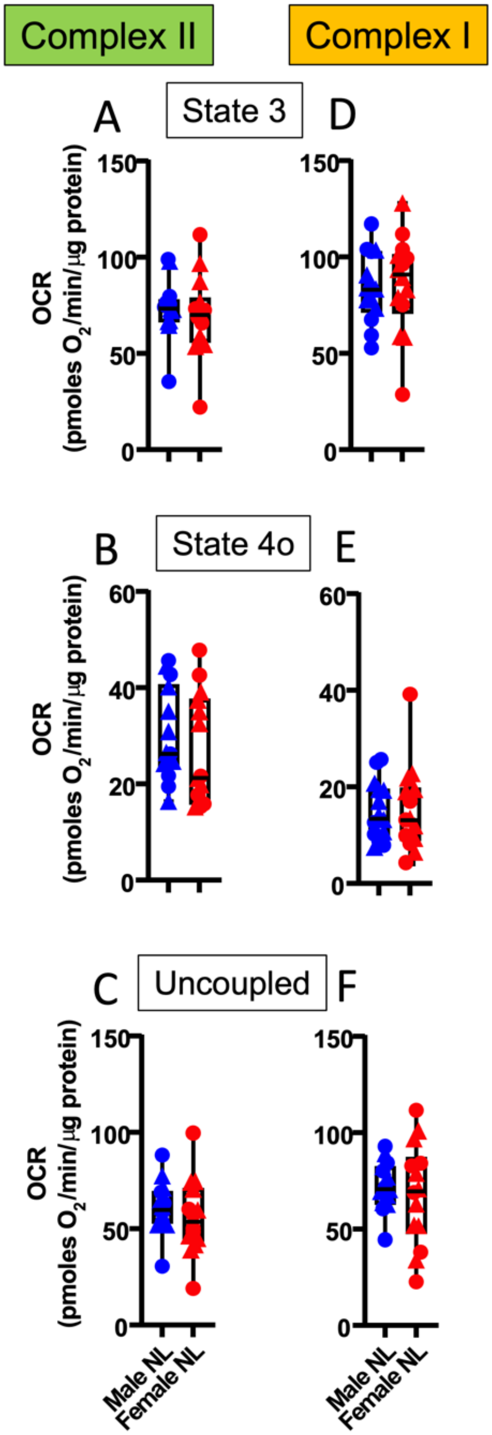


**Supplemental Figure 6. No sex differences in absolute oxygen consumption in cortical permeabilized synaptosomes derived from healthy control males and females. (A – D)** Quantitative analysis of mitochondrial OCR for State 3 (A and D), State 4o (B and E), and Uncoupled (C and F) parameters in Complex II mediated (A – C) and Complex I mediated (D – F) respirometry using permeabilized cortical synaptosomes from control (NL); male mice (blue) and female mice (red). Data shown from two separate experiments (circle symbol: 1st experiment, triangle symbol: 2nd experiment). n=14 per group.
